# Quantification and accuracy of clinical [11C]-PiB PET/MRI: the effect of MR-based attenuation correction

**DOI:** 10.1186/2197-7364-1-S1-A69

**Published:** 2014-07-29

**Authors:** Ian Law, Flemming L Andersen, Adam E Hansen, Steen G Hasselbalch, Claes Ladefoged, Sune H Keller, Søren Holm, Liselotte Højgaard

**Affiliations:** The Department of Clinical Physiology, Nuclear Medicine and PET, Rigshospitalet, Kragujevac, Denmark; Memory Disorders Research Group, Rigshospitalet, Kragujevac, Denmark

The Dixon-Water-Fat segmentation (DWFS) method is a standard attenuation correction (AC) method in PET/MRI on the Siemens mMR and has demonstrated a systematic quantitative bias in [18F]-FDG-PET/MRI studies of the brain compared to PET/CT. The aim of this study was to evaluate the impact of DWFS-AC in a hybrid PET/MR scanner on regional and global quantitation of [11C]-PiB cerebral amyloid imaging of the brain and in the clinical reading.

Twenty-eight healthy volunteers, 6 with mild cognitive impairment (MCI), 4 with Alzheimers disease (AD), and 6 other dementia cases underwent a simultaneous PET/MRI (Siemens mMR) acquisition 40 min pi of (170-709) MBq [11C]-PiB. A single 30 min frame was reconstructed twice (OSEM-3D, 21it 4 sub, 5 mm Gauss) with the only difference being the AC calculation. AC was performed using either the standard DWFS or a head low-dose CT scan acquired independently on the same day. Activity concentration (Bq/mL) was sampled on AC-PET from symmetrically delineated ROI’s. Ratios of region-to-cerebellar grey matter (SUVr) were calculated. Amyloid uptake was considered abnormal at cortical SUVr >1.5 or increased amyloid uptake in two or more grey matter regions on visual evaluation.

The average activity concentration in all ROI’s was biased by -18 % in DWFS-AC compared to CT-AC. Although the visual categorization of both, amyloid-positive (n=11) and -negative (n=33) scans was unaffected by DWFS-AC, 3 healthy subjects were quantitatively reclassified as amyloid positive. The SUVr values were overestimated by 0.11 in the caudate nuclei and 0.04 in lateral cortical ROI’s in DWFS-AC compared to CT-AC.

The visual and quantitative consequences of MR-AC using DWFS in the brain with [11C]-PiB exhibit a noticeable radially variable bias. Although robust in visual evaluation, the quantitative diagnostic criteria (SUVr) using this biomarker with DWFS-AC may need to be modified.

